# Prevalence of psychological distress and associated factors in tuberculosis patients in public primary care clinics in South Africa

**DOI:** 10.1186/1471-244X-12-89

**Published:** 2012-07-27

**Authors:** Karl Peltzer, Pamela Naidoo, Gladys Matseke, Julia Louw, Gugu Mchunu, Bomkazi Tutshana

**Affiliations:** 1 HIV/STI and TB (HAST) Research Programme, Human Sciences Research Council, Pretoria, South Africa; 2 Department of Psychology, University of the Free State, Bloemfontein, South Africa; 3 Department of Psychology, University of the Western Cape, Cape Town, South Africa

## Abstract

**Background:**

Psychological distress has been rarely investigated among tuberculosis patients in low-resource settings despite the fact that mental ill health has far-reaching consequences for the health outcome of tuberculosis (TB) patients. In this study, we assessed the prevalence and predictors of psychological distress as a proxy for common mental disorders among tuberculosis (TB) patients in South Africa, where over 60 % of the TB patients are co-infected with HIV.

**Methods:**

We interviewed 4900 tuberculosis public primary care patients within one month of initiation of anti-tuberculosis treatment for the presence of psychological distress using the Kessler-10 item scale (K-10), and identified predictors of distress using multiple logistic regressions. The Kessler scale contains items associated with anxiety and depression. Data on socio-demographic variables, health status, alcohol and tobacco use and adherence to anti-TB drugs and anti-retroviral therapy (ART) were collected using a structured questionnaire.

**Results:**

Using a cut off score of ≥28 and ≥16 on the K-10, 32.9 % and 81 % of tuberculosis patients had symptoms of distress, respectively. In multivariable analysis older age (OR = 1.52; 95 % CI = 1.24-1.85), lower formal education (OR = 0.77; 95 % CI = 0.65-0.91), poverty (OR = 1.90; 95 % CI = 1.57-2.31) and not married, separated, divorced or widowed (OR = 0.74; 95 % CI = 0.62-0.87) were associated with psychological distress (K-10 ≥28), and older age (OR = 1.30; 95 % CI = 1.00-1.69), lower formal education (OR = 0.55; 95 % CI = 0.42-0.71), poverty (OR = 2.02; 95 % CI = 1.50-2.70) and being HIV positive (OR = 1.44; 95 % CI = 1.19-1.74) were associated with psychological distress (K-10 ≥16). In the final model mental illness co-morbidity (hazardous or harmful alcohol use) and non-adherence to anti-TB medication and/or antiretroviral therapy were not associated with psychological distress.

**Conclusions:**

The study found high rates of psychological distress among tuberculosis patients. Improved training of providers in screening for psychological distress, appropriate referral to relevant health practitioners and providing comprehensive treatment for patients with TB who are co-infected with HIV is essential to improve their health outcomes. It is also important that structural interventions are promoted in order to improve the financial status of this group of patients.

## Background

South Africa has 0.7 % of the world’s population and 28 % of the world’s population of HIV and TB co-infected individuals. It has been estimated that there is approximately 60 % of people with TB who are co-infected with HIV [1]. Co-infected patients have almost double the chance of contracting multiple drug resistant TB (MDR-TB) as well as extra drug resistant TB (XDR-TB). These patients also have a high mortality rate due to co-infection with HIV [2].

Common mental disorders (CMDs), which include depression, anxiety and somatoform disorders, make a significant contribution to the burden of disease and disability in low- and middle-income countries (LMICs) [3,4].These conditions are responsible for up to 10 % of the total global disease burden [5]. At least one-third of all patients seen in primary care facilities in LMICs present with CMDs. CMDs are not recognised nor effectively treated in a majority of these patients [5]. Although depressive and anxiety disorders are classified as separate diagnostic categories in the ICD-10 [6], the concept of CMDs is valid for public health interventions owing to the high degree of co-morbidity between these disorders in primary care and the similarity in epidemiological profiles and treatment responsiveness [7].

Several authors found frequent comorbidity of TB and common mental disorders [8,9] . Few studies have investigated common mental disorders in TB patients in low and middle income countries (LMICs) and have found high rates of CMDs in Pakistan 46.3 %-80 % [10-12], Nigeria 27.7 %-30 % [13,14], Ethiopia 64 % [15], India 76 % [16], South Africa 46 % [17,18] and Turkey 19 %-26 % [19].

Factors associated with CMDs in TB patients included: male gender [11], older age groups, the young and the elderly [11,13,14], low educational attainment [14], financial status, no source of income [13,15], in day labourers: [15] an increase in the number of symptoms reported, more serious perceived consequences and less control over their illness [10,13], in TB/HIV co-infected [15] individuals : perceived stigma [15], poor perceived health status [15], adverse effect on drug compliance and TB treatment [11,20] and negative TB treatment outcome (death) [21].

The aim of this study was to establish the prevalence and associated factors of common mental disorders (namely anxiety and depression) in tuberculosis public primary care patients in South Africa.

## Methods

### Study design

This is a cross-sectional survey of tuberculosis patients in public primary care clinics in South Africa.

### Sample and procedure

Three provinces in South Africa with the highest TB caseload were selected for inclusion in the study. One district in each province (N = 3) with the highest TB caseloads were ultimately included. These districts were Siyanda in Northern Cape Province, Nelson Mandela Metro in the Eastern Cape Province and eThekwini in KwaZulu-Natal Province. Within each of these three study districts 14 public primary health care facilities were selected on the basis of the highest TB caseloads per clinic (N = 42). The type of health facilities were primary health care clinic or community health centre. All new TB and new retreatment patients were consecutively interviewed within one month of initiation of anti-tuberculosis treatment. The interview was conducted by trained external research assistants for a period of 6 months from mid- April 2011 to mid- October 2011 in all 42 clinics. A health care provider who identified a new TB treatment or retreatment patient (within one month of initiating treatment) and 18 years and older informed the patient about the study and referred the patient for participation if they were willing. A research assistant obtained informed consent from these patients attending the primary care facility to participate in the interview. Questionnaires were translated and back translated into the major languages of the study participants (Afrikaans, Tswana, Xhosa and Zulu). Ethical approval was obtained from the Human Sciences Research Council Research Ethics Committee (Protocol REC No.1/16/02/11) and the Department of Health in South Africa.

### Measures

#### Socioeconomic characteristics

A researcher-designed questionnaire was used to record information on participants’ age, gender, educational level, marital status, income, employment status, dwelling characteristics and residential status. *Poverty* was assessed by 5 pertinent items on the questionnaire by asking about the availability or non-availability of shelter, fuel or electricity, clean water, food and cash income in the past week. Response options ranged from 1 = “Not one day” to 4 = “Every day of the week”. Participants ranked high on poverty if they had higher scores on non-availability of essential items. The total score ranged from 5 to 20: 5 = being low on poverty, 6-12 = medium level of poverty and 13-20 = high levels of poverty. The Cronbach alpha for the poverty index in this study was 0.89.

The *Kessler Psychological Distress Scale* (K-10) was used to measure global psychological distress, including significant pathology which does not meet formal criteria for a psychiatric illness [22,23]. This scale measures the following symptoms over the preceding 30 days by asking: “In the past 30 days, how often did you feel: nervous; so nervous that nothing could calm you down; hopeless; restless or fidgety; so restless that you could not sit still; depressed; that everything was an effort; so sad that nothing could cheer you up; worthless; tired out for no good reason?” The frequency with which each of these items was experienced was recorded using a five-point Likert scale ranging from “none of the time” to “all the time”. These scores were then added with increasing total scores reflecting an increasing degree of psychological distress. The K-10 has been shown to capture variability related to non-specific depression and anxiety but does not measure suicidality or psychoses [24]. This scale serves to identify individuals who are likely to meet formal definitions for anxiety and/or depressive disorders, as well as to identify individuals with sub-clinical illness who may not meet formal definitions for a specific disorder [22]. This scale is increasingly used in population mental health research and has been validated in multiple settings [25] including HIV positive individuals in South Africa [26] and a population-based survey in South Africa [27]. There was significant agreement among HIV patients between the K-10 and the MINI-defined depressive and anxiety disorders, with the best screening cut off score of 28 [26]. A receiver operating characteristic (ROC) curve analysis indicated that the K-10 showed agreeable sensitivity and specificity in detecting depression (area under the ROC curve, 0.77), generalized anxiety disorder (0.78), and posttraumatic stress disorder (PTSD) (0.77), with the best cut off of 28 [27]. Further, the K10 demonstrated moderate discriminating ability in detecting depression and anxiety disorders in the general population in South Africa; evidenced by area under the receiver operating curves of 0.73 and 0.72 respectively, with a cut off of 16 [27]. We examined the K-10 scale using two cut offs (16 and 28), as found in validation studies in South Africa [26,27]. The internal reliability coefficient for the K-10 in this study was alpha = 0.92.

#### Alcohol consumption

The 10-item Alcohol Disorder Identification Test (AUDIT) [28] assesses alcohol consumption level (3 items), symptoms of alcohol dependence (3 items), and problems associated with alcohol use (4 items). Heavy episodic drinking is defined as the consumption of six standard drinks (10 g alcohol) or more on a single occasion [28]. In South Africa a standard drink has 12 g of alcohol. Because the AUDIT is reported to be less sensitive at identifying risk drinking in women [29], the cut-off point for binge drinking among women (4 units) was reduced by one unit as compared to men (5 units), as recommended by Freeborn et al. [29]. Responses to items on the AUDIT are rated on a 4-point Likert scale from 0 to 4, for a maximum score of 40 points. Higher AUDIT scores indicate more severe levels of risk; scores 8 indicate a tendency to problematic drinking. The Alcohol Use Disorders Identification Test (AUDIT) was developed by the World Health Organization as an effective screening instrument for alcohol use problems among patients seeking primary care for other medical problems in international settings including African countries (Kenya and Zimbabwe) [28,30] and has been validated in HIV patients in South Africa showing excellent sensitivity and specificity in detecting MINI-defined dependence/abuse (area under the receiver-operating characteristic curve, 0.96) [31] and among TB and HIV patients in primary care in Zambia demonstrating good discriminatory ability in detecting MINI-defined current AUDs (AUDIT = 0.98 for women and 0.75 for men) [32]. Cronbach alpha for the AUDIT in this sample was 0.92, indicating excellent reliability. Hazardous drinking is defined as a quantity or pattern of alcohol consumption that places patients at risk for adverse health events, while harmful drinking is defined as alcohol consumption that results in adverse events (e.g. physical or psychological harm) [33].

#### Tobacco use

Two questions were asked about the use of tobacco products, namely 1) Do you currently use one or more of the following tobacco products (cigarettes, snuff, chewing tobacco, cigars, etc.)? with a response option of “yes” or “no” and 2) In the past month, how often have you used one or more of the following tobacco products (cigarettes, snuff, chewing tobacco, cigars, etc.)? with the response options of once or twice, weekly, almost daily and daily. Current tobacco use was defined as having used any tobacco in the past month.

#### Perceived general health

In order to ascertain participant’s perceived general health they were asked,: In general, would you say your health is: excellent, very good, good, fair or poor? This measure was categorized based on participant response (very good = excellent/very good, good, and poor = fair/poor).

TB treatment status, HIV status and antiretroviral treatment was assessed by self-report and from medical information. Anti TB medication adherence was assessed with the question “In your tuberculosis treatment in the past 3–4 weeks how many percent were you taking your anti-tuberculosis medication?” using the Visual Analogue Scale. TB medication non-adherence was defined as having taken less than 90 percent of their anti-tuberculosis medication. Antiretrovitral Therapy (ART) adherence was assessed with the question “How many percent of your HIV medication did to take in the past 4 weeks?” using the Visual Analogue Scale. ART non-adherence was defined as having taken less than 90 percent of ART.

### Data analysis

Data were analyzed using Statistical Package for the Social Sciences (SPSS) for Windows software application programme version 19.0. Frequencies, means, standard deviations, were calculated to describe the sample. Data were checked for normality distribution and outliers. Interaction between predictor variables was also examined and it was found that none of the variables had a Variance Inflation Factor (VIF) value above 2.5. Associations of psychological distress and harmful alcohol use were identified using logistic regression analyses. Following each univariate regression, multivariable regression models were constructed. Independent variables from the univariate analyses were entered into the multivariable model if significant at P < 0.05 level. For each model, the R^2^ is presented to describe the amount of variance explained by the multivariable model. Probability below 0.05 was regarded as statistically significant.

## Results

### Sample characteristics and psychological distress

From the total sample (N = 4935) included in the study 35 (0.7 %) refused to participate. As a result of the refusals the final sample comprised 4900. More than half (54.5 %) of the participants were men and 45.5 % were women, with a mean age of 36.2 years (SD = 11.5) and the age range was 18 years to 93 years. Almost four-fifth of the participants (78.4 %) were between 18 to 44 years old, the majority (72.7 %) was never married, 27.7 % had completed secondary education, and 17 % scored high on the poverty index. The largest population group of the participants was Black African (84.6 %), followed by individuals of mixed race (Coloured: derived from Asian, European, and Khoisan and other African ancestry) (13.1 %) and Indian , Asian or White (2.3 %). 76.6 % of the total sample were new TB patients and 23.4 % were retreatment TB patients. Of those particpants that tested for HIV 59.9 % were HIV positive, 22.1 % of the HIV positive patients were on ART. 9.6 % had never tested for HIV. One in five patients (20 %) were daily or almost daily tobacco users, 23.3 % were hazardous or harmful alcohol users (AUDIT 8 or more) and 46.3 % perceived their health status as fair or poor. Regarding adherence to TB medication, 33.9 % indicated that they had missed at least 10 % their medication in the last 3–4 weeks. From those who were on antiretroviral treatment, 42.1 % reported that they had at least 10 % of their ARVs in the last 4 weeks. The overall prevalence of psychological distress in this study was 32.9 % (K-10 ≥28) and 81.1 % (K-10 ≥16), respectively (see Table 1). Of those who screened positive for psychological distress (anxiety/depression) (K-10 ≥28), 8.3 % were using anti-depression medication currently. Those using anti-depression medication were not more likely to screen positive for anxiety/depression (8.7 % vs. 6.5 %; χ^2^ = 1.36, P = 0.244).

**Table 1:** Sample characteristics

**Socio-demographics**	**Total**		**Psychological distress (≥28)**		**Psychological distress (≥16)**	
	**N**	**%**	**N**	**%**	**N**	**%**
*All*	4900		1614	32.9	3970	81.0
*Gender*						
Male	2631	54.5	888	33.8	2127	80.8
Female	2194	45.5	706	32.2	1786	81.4
*Age*						
18-30	1769	36.6	525	29.7	1389	78.5
31-44	2078	41.8	663	32.9	1636	81.1
45 or more	1040	21.5	396	38.1	886	85.2
*Marital status*						
Never married	3323	72.7	1127	33.6	2723	81.1
Married/cohabitating	982	21.5	298	29.8	785	78.4
Separated/divorced/widowed	265	5.8	102	37.1	235	85.5
Never married	3323	72.7	1127	33.6	2723	81.1
Married/cohabitating	982	21.5	298	29.8	785	78.4
Separated/divorced/widowed	265	5.8	102	37.1	78.4	85.5
	*Education*						
Grade 7 or less	1269	26.3	487	38.4	1091	86.0	
Grade 8-11	2213	45.9	683	30.9	1804	81.5	
Grade 12 or more	1336	27.7	418	31.3	1000	74.9	
*Poverty index (5–20)*							
Low (5)	1592	35.0	422	26.1	1238	76.6	
Medium (6–12)	2195	48.2	757	34.0	1782	80.0	
High (13–20)	768	16.9	334	43.0	698	89.9	
*Population group*							
Black African	4078	84.6	1402	34.4	3322	81.5	
Coloured	634	13.1	158	24.9	488	77.0	
Indian/Asian/White or other	114	2.3	35	31.5	89	80.2	
**Health variables**							
New TB patient	3650	76.6	1219	32.9	2971	80.1	
Retreatment TB patient	1113	23.4	380	33.7	944	83.7	
HIV positive	2585	59.9	874	33.4	2175	83.0	
HIV negative	1728	40.1	555	31.6	1370	77.9	
HIV unknown	385	9.6	143	37.1	312	81.0	
Partner HIV positive	1192	27.2	427	35.3	1010	83.5	
Partner HIV negative or unknown status	3194	72.8	1067	32.9	2590	79.8	
Daily or almost daily tobacco use	980	20.0	363	37.0	795	81.1	
Hazardous or harmful alcohol use	1120	23.3	380	33.9	941	84.0	
*Perceived health status*							
Excellent/very good	912	19.1	350	37.7	746	80.4	
Good	1646	34.6	388	23.3	1244	74.6	
Fair/poor	2205	46.3	870	38.9	1916	85.6	
On antiretroviral therapy	974	22.1	342	37.7	769	84.9	
***Adherence***							
Non-adherence to TB treatment	1138	33.9	368	32.3	903	79.3	
Non-adherence to ART	410	42.1	170	41.5	341	83.4	

### Predictors of psychological stress

In univariate analysis older age, lower formal education, poverty, not married, separated, divorced or widowed and daily or almost daily tobacco use were associated with psychological distress (K-10 ≥28). In multivariable analysis older age (OR = 1.52; 95 % CI = 1.24-1.85), lower formal education (OR = 0.77; 95 % CI = 0.65-0.91), poverty (OR = 1.90; 95 % CI = 1.57-2.31) and not married, separated, divorced or widowed (OR = 0.74; 95 % CI = 0.62-0.87) were associated with psychological distress (K-10 ≥28). Further, in univariate analysis older age, lower formal education, poverty, being a retreatment TB patient, being HIV positive, partner being HIV positive and hazardous or harmful alcohol use were associated with psychological distress (K-10 ≥16). In multivariable analysis older age (OR = 1.30; 95 % CI = 1.00-1.69), lower formal education (OR = 0.55; 95 % CI = 0.42-0.71), poverty (OR = 2.02; 95 % CI = 1.50-2.70) and being HIV positive (OR = 1.44; 95 % CI = 1.19-1.74) were associated with psychological distress (K-10 ≥16). In the final model mental illness co-morbidity (hazardous or harmful alcohol use) and non-adherence to anti-TB medication and/or antiretroviral therapy were not associated with psychological distress (see Table 2).

**Table 2:** Association of socioeconomic and clinical characteristics and psychological distress among TB patients in 42 clinics, South Africa, 2011

	**K-10 (28 or more)**		**K-10 (16 or more)**	
	**Crude OR**	**Adjusted OR**	**Crude OR**	**Adjusted OR**
**Socio-demographics**	**(95 % CI)**^**a**^	**(95 % CI)**^**a,b**^	**(95 % CI)**^**a**^	**(95 % CI)**^**a,c**^
*Age*				
18-30	1.00	1.00	1.00	1.00
31-44	1.16 (1.01-1.33)*	1.20 (1.03-1.40)*	1.17 (1.00-1.37)	1.04 (0.86-1.26)
45 or more	1.46 (1.24-1.71)***	1.52 (1.24-1.85)***	1.57 (1.28-1.93)***	1.30 (1.00-1.69)*
*Gender*				
Female	1.00	1.00	1.00	1.00
Male	1.07 (0.95-1.21)	1.06 (0.92-1.21)	0.96 (083–1.11)	0.90 (0.75-1.07)
*Marital status*				
Not married	1.00	1.00	1.00	---
Married/cohabitating	0.84 (0.72-0.98)*	0.74 (0.62-0.87)***	0.85 (0.71-1.01)	
Separated/divorced/widowed	1.17 (0.90-1.51)	1.05 (0.36-3.91)	1.37 (0.97-1.93)	
*Education*				
Grade 7 or less	1.00	1.00	1.00	1.00
Grade 8-11	0.72 (0.62-0.83)***	0.77 (0.65-0.91)**	0.72 (0.59-0.87)***	0.78 (0.62-0.99)*
Grade 12 or more	0.73 (0.62-0.86)***	0.85 (0.70-1.02)	0.49 (0.40-0.59)***	0.55 (0.42-0.71)***
*Poverty*				
Low	1.00	1.00	1.00	1.00
Medium	1.46 (1.27-1.68)***	1.39 (1.20-1.62)***	1.23 (1.05-1.43)**	1.07 (0.86-1.33)
Poverty high	2.14 (1.78-2.56)***	1.90 (1.57-2.31)***	2.74 (2.11-3.56)***	2.02 (1.50-2.70)***
**Health characteristics**				
New TB patient	1.00	---	1.00	1.00
Retreatment TB patient	1.04 (0.84-1.11)		1.27 (1.06-1.52)**	1.19 (0.96-1.48)
TB/HIV co-infected	1.09 (0.96-1.24)	---	1.39 (1.20-1.62)***	1.44 (1.19-1.74)***
Partner HIV positive vs. negative or unknown	1.11 (0.97-1.28)	---	1.28 (1.08-1.53)**	1.14 (0.93-1.41)
Daily/almost daily tobacco use	1.26 (1.09-1.45)**	1.06 (0.89-1.25)	1.01 (0.84-1.21)	---
Hazardous or harmful alcohol use	1.05 (0.91-1.21)	---	1.33 (1.11-1.59)**	1.05 (0.83-1.31)
*Adherence*				
Non-ART adherence	1.22 (0.94-1.59)	---	1.84 (0.70-4.80)	---
TB non-adherence	0.92 (0.79-1.07)	---	0.90 (0.75-1.07)	---

^a^ Using “enter” LR selection of variables.

^b^ Hosmer and Lemeshow Chi-square 12.97, df 8, 0.113; Cox and Snell R^2^ 0.02; Nagelkerke R^2^ 0.03.

^c^ Hosmer and Lemeshow Chi-square 3.47, df 8, 0.902; Cox and Snell R^2^ 0.03; Nagelkerke R^2^ 0.04.

* P < 0.05; ** P < 0.01; *** P < 0.001.

## Discussion

A high overall prevalence of psychological distress (32.9 % and 81 % according to the K-10 score ≥28 and K-10 score ≥16, respectively) was found in this large sample of tuberculosis public primary care patients in South Africa. Caseness of psychological distress or common mental disorder was assessed using two different cut-offs (K-10 score ≥28 and K-10 score ≥16), as found in two different previous validation studies in South Africa [26,27]. The uncertainty regarding the correct K-10 cut-off for this study group is a major limitation of this study.

Several studies showed that the K-10 had good psychometric properties [25,26] and can discriminate between cases and non-cases reported in the Diagnostic and Statistical Manual of Mental Disorders (DSM-IV) [15,34]. The finding of 32.9 % to 81 % (using cut off scores of 28 and 16 respectively) the prevalence of psychological distress in this study is in line with the prevalence rates of depression or common mental disorders in most other studies with tuberculosis patients 46 %-80 % in LMICs [10-12,15-18]. The differences in prevalence of common mental disorders in the studies cited in the literature could be attributable to several factors including the population being studied, the study periods during the TB treatment course and the diagnostic tools used [15]. It is possible that increased rates of psychological distress were found in this study because the assessment followed within a short time (within one month) of the TB diagnosis which might not have persisted at a later stage of the disease course or upon completion of TB treatment. Andersen et al. [27] have suggested that the Kessler scales had significantly lower discriminating ability in regard to depression and anxiety disorders among the Black African than among the combined non-Black African population group in South Africa, and they attribute this to differential item biased measurement. The Black African population group is usually highly represented among the lowest socioeconomic status groups in South Africa and often lack basic necessities as compared to the other population groups in South Africa. Consequently, they may more likely endorse K-10 items such as “How often do you feel that everything is an effort?”[27] In this study, however, there were no significant differences in psychological distress rates between Black African, Indian/Asian or Whites population groups. Spiess et al. [26] also did not find a significant difference in validity of the K-10 in detecting depression and anxiety disorders among HIV infected South Africans across gender, age, education, or ethnicity categories.

Given the relatively large difference between the levels of psychological distress using a cut-off score of 28 versus 16 on the K-10 in this study, it is important to consider which cut-off score is more appropriate for use in a clinical setting within the public health sector. The authors in this study recommend the use of a cut-off score of 16 for use in South Africa, particularly within the public sector health clinics in order for cost-efficient treatment programmes to be implemented on a large scale.

In multivariable analysis the study found that lower formal education and poverty were associated with psychological distress. Low socioeconomic status has also been found in other studies to be associated with common mental disorders in TB patients [13-15]. Most studies in developing countries showed an association between indicators of poverty and the risk of mental disorders, the most consistent association being with low levels of education [3,35,36]. Many patients in low and middle income countries suffer from common mental disorders because of the stress caused by poverty [3,15,37] and associated factors such as the experience of insecurity and hopelessness, rapid social change and the risks of violence and physical ill-health may explain the greater vulnerability of the poor to common mental disorders [34]. Financial empowerment of patients may reduce their levels of depression, and improve the compliance rate to anti-TB medication which could ultimately result in an improved quality of life [13].

Older age in this study was associated with psychological stress. This finding is consistent with the findings of other studies among TB patients [13,14] but was not consistent with findings in general population studies [38]. . This increased prevalence of distress in older participants may be due to increased responsibilities such as child care, care of other family members, employment and economic responsibilities, having to cope with chronic illness conditions, including HIV in the older age group [13]. Marital status (being married or cohabitating) has been found in this study to serve a protective factor from psychological stress (K-10 ≥28) and this finding is consistent with the findings of other studies [39,40]. Being married or cohabitating provides social support thereby reducing the levels of psychological distress [40]. In contrast to some studies [11,13] but in agreement with other studies [41], we did not find a significant association between gender and psychological distress.

Being TB/HIV co-infected was found in this study to be associated with a higher rate of common mental disorders (using the K-10 cut off of ≥16) than among non-coinfected patients, a finding consistent with other studies [15]. Being diagnosed with HIV which is a terminal life-long disease associated with high levels of stigma may also lead to high rates of mental disorder [42]. Substance use (hazardous or harmful alcohol use and current tobacco use) in this study was associated with psychological distress. This finding is similar to the findings in other studies which found substance use to be associated with depression and psychological distress in general patient population groups [43-46]. Daily or almost daily tobacco use was also found in this study to be associated with psychological distress a finding similar to the findings of other studies [43]. In this study there was no association found between TB and HIV treatment non-adherence and common mental disorders as found in other studies [11,15,20,47]. It is possible that the impact of psychological stress on treatment adherence may take longer to take effect than at the beginning of TB treatment.

### Study limitations

Caution should be taken when interpreting the results of this study because of certain limitations. Generalisability of our findings is limited to TB and HIV patients on treatment in public primary care centres in South Africa. Furthermore, measures of anxiety and depression at a general population level in South Africa may be needed so that the diagnostic accuracy of common mental disorders among patients with anxiety and depression can be compared to those without these disorders. This study was a cross-sectional one which implies that no statement of causality between the variables can be made. A further limitation was that most variables were assessed by self-report and socially desirable responses may have been given. The Kessler 10 scale is not 100 % sensitive and specific which may have resulted in misdiagnosis or missed cases of common mental disorder. Some areas of assessment such as number/severity of symptoms reported, illness perceptions [10,13,47] and perceived stigma [15] were not included in the study. These factors are known to be related to common mental disorder in TB patients.

## Conclusion

The study found high rates of psychological distress among tuberculosis patients. Improved training of providers in screening for psychological distress, referral and psychotropic and/or psychological intervention treatment plans for adult patients with anxiety, depression or mixed common mental health problems are indicated [43]. Accurate diagnosis of co-morbid depressive and anxiety disorders in patients with chronic medical illness is essential in understanding the cause and optimising the management of somatic symptom burden [44]. In addition, measures to reduce psychological distress among patients with TB should include a more comprehensive care approach which should involve sectors outside of health that are responsible for structural adjustment programmes which will ultimately improve the financial status of this group of patients. Ultimately structural adjustments through government economic policies to alleviate poverty combined with psychological interventions are needed to improve the health outcomes of TB patients and those co-infected with HIV.

## Competing interests

The authors declare that they have no competing interests.

## Authors’ contributions

**KP** conceived and designed the study, analyzed the data and drafted the manuscript. **PN** and **GM** participated in the design, conception and reviewed the article. **JL, GM** and **BT** were involved in report writing and reviewing. All authors read and approved the final manuscript.

## Pre-publication history

The pre-publication history for this paper can be accessed here:

http://www.biomedcentral.com/1471-244X/12/89/prepub

## References

[B1] World Health Organisation (WHO) Global TB control report 2010 WHO, Geneva, Switzerland 2010

[B2] Department of Health Tuberculosis strategic plan for South Africa 2007–2011 2007 Government Printers, Pretoria

[B3] World Health Organization World Health Report 2001, Mental Health: New Understanding, New Hope 2001 WHO, Geneva

[B4] Lopez DA Mathers DC Ezzati M Jamison TD Murray JLC Global Burden of Disease and Risk Factors 2006 Oxford University Press/World Bank, New York

[B5] Siddiqi K Siddiqi N Treatment of common mental disorders in primary care in low- and middle-income countries Trans R Soc Trop Med Hyg 2007 101 10 957 958 10.1016/j.trstmh.2007.04.006 17540425

[B6] World Health Organization International Statistical Classification of Diseases and Related Health Problems. 10th revision (ICD-10), version for 2007 2007 WHO, Geneva

[B7] Patel V Simon G Chowdhary N Kaaya S Araya R Packages of care for depression in low- and middle-income countries PLoS Med 2009 6 10 e1000159 10.1371/journal.pmed.1000159 19806179 PMC2747016

[B8] Trenton AJ Currier GW Prim Care Companion J Clin Psychiatry 2001 3 6 236 243 10.4088/pcc.v03n0610 PMC181192 15014591

[B9] Yang L Wu DL Guo HG Liu JW A study of the psychological and social factors in patients with pulmonary tuberculosis Zhonghua Jie He He Hu Xi Za Zhi 2003 26 11 704 707 14703449

[B10] Hussain MO Dearman SP Chaudhry IB Rizvi N Waheed W The relationship between anxiety, depression and illness perception in tberculosis patients in Pakistan Clin Pract Epidemiol Ment Health 2008 26 4 4 10.1186/1745-0179-4-4 PMC2288599 18302758

[B11] Sulehri MA Dogar IA Sohail H Mehdi Z Azam M Niaz O Javed MS Sajjad IA Iqbal Z Prevalence of Depression Among Tuberculosis Patients A.P.M.C 2010 4 2 133 137

[B12] Aamir SA Co-morbid anxiety and depression among pulmonary tuberculosis patients J Coll Physicians Surg Pak 2010 20 10 703 704 20943121 10.2010/JCPSP.703704

[B13] Issa BA Yussuf AD Kuranga SI Depression comorbidity among patients with tuberculosis in a university teaching hospital outpatient clinic in Nigeria Mental Health Family Med 2009 6 133 138 PMC2838651 22477903

[B14] Aghanwa HS Erhabor GE Demographic/socioeconomic factors in mental disorders associated with tuberculosis in southwest Nigeria J Psychosom Res 1998 45 4 353 360 10.1016/S0022-3999(98)00006-3 9794281

[B15] Deribew A Tesfaye M Hailmichael Y Apers L Abebe G Duchateau L Colebunders R Common mental disorders in TB/HIV co-infected patients in Ethiopia BMC Infect Dis 2010 10 201 10.1186/1471-2334-10-201 20618942 PMC2911449

[B16] Prakash C Sangita S Study of Psychiatric co - morbidity in cases of tuberculosis patients undergoing treatment Indian J Public Health Res Development 2011 2 2 111 113

[B17] Westaway MS Wolmarans L Depression and self-esteem: rapid screening for depression in black, low literacy, hospitalized tuberculosis patients Soc Sci Med 1992 35 10 1311 1315 10.1016/0277-9536(92)90184-R 1439914

[B18] Naidoo P Mwaba K Helplessness, depression and social support among TB patients at a public health site: a prevalence study J Soc Beh Pers 2010 38 10 1323 1334 10.2224/sbp.2010.38.10.1323

[B19] Aydin IO Uluşahin A Depression, anxiety comorbidity, and disability in tuberculosis and chronic obstructive pulmonary disease patients: applicability of GHQ-12 Gen Hosp Psychiatry 2001 23 2 77 83 10.1016/S0163-8343(01)00116-5 11313075

[B20] Shin S Muñoz M Espiritu B Zeladita J Sanchez E Callacna M Rojas C Arevalo J Wu Y Caldas A Sebastian JL Psychosocial impact of poverty on antiretroviral nonadherence among HIV-TB coinfected patients in Lima, Peru J Int Assoc Physicians AIDS Care 2008 7 2 74 81 10.1177/1545109708315326 18319510

[B21] Duarte EC Bierrenbach AL Barbosa da Silva J Tauil PL de Fátima Duarte E Factors associated with deaths among pulmonary tuberculosis patients: a case-control study with secondary data J Epidemiol Community Health 2009 63 3 233 238 10.1136/jech.2008.078972 19066188

[B22] Kessler R Andrews G Colpe LJ Hiripi E Mroczek DK Normand ST Walters EE Zaslavsky AM Short screening scales to monitor population prevalences and trends in nonspecific psychological distress Psychol Med 2002 32 959e976 12214795 10.1017/s0033291702006074

[B23] Kessler RC Barker PR Colpe LJ Epstein JF Gfroerer JC Hiripi E Howes MJ Normand SL Manderscheid RW Walters EE Zaslavsky AM Screening for serious mental illness in the general population Arch Gen Psychiatry 2003 60 2 184e 189e 10.1001/archpsyc.60.2.184 12578436

[B24] Brooks RT Beard J Steel Z Factor structure and interpretation of the K10 Psychol Assess 2006 18 1 62e 70e 16594813 10.1037/1040-3590.18.1.62

[B25] Andrews G Slade T Interpreting scores on the Kessler Psychological Distress Scale (K10) Aust N Z J Public Health 2001 25 494 497 10.1111/j.1467-842X.2001.tb00310.x 11824981

[B26] Spies G Kader K Kidd M Smit J Myer L Stein DJ Seedat S Validity of the K-10 in detecting DSM-IV-defined depression and anxiety disorders among HIV-infected individuals AIDS Care 2009 21 9 1163 1168 10.1080/09540120902729965 20024776

[B27] Andersen LS Grimsrud A Myer L Williams DR Stein DJ Seedat S The psychometric properties of the K10 and K6 scales in screening for mood and anxiety disorders in the South African Stress and Health study Int J Methods Psychiatr Res 2011 20 4 215 223 10.1002/mpr.351 22113964 PMC6878543

[B28] Babor TF Higgins-Biddle JC Brief intervention for hazardous and harmful drinking a manual for use in primary care 2001 World Health Organziation, Geneva, Switzerland

[B29] Freeborn DK Polen MR Hollis JF Senft RA Screening and brief intervention for hazardous drinking in an HMO: effects on medical care utilization J Behav Health Serv Res 2000 27 4 446 453 10.1007/BF02287826 11070638

[B30] Saunders JB Aasland OG Amundsen A Grant M Alcohol consumption and related problems among primary health care patients: WHO collaborative project on early detection of persons with harmful alcohol consumption-I Addiction 1993 88 3 349 362 10.1111/j.1360-0443.1993.tb00822.x 8461852

[B31] Myer L Smit J Roux LL Parker S Stein DJ Seedat S Common mental disorders among HIV-infected individuals in South Africa: prevalence, predictors, and validation of brief psychiatric rating scales AIDS Patient Care STDS 2008 22 2 147 158 10.1089/apc.2007.0102 18260806

[B32] Chishinga N Kinyanda E Weiss HA Patel V Ayles H Seedat S Validation of brief screening tools for depressive and alcohol use disorders among TB and HIV patients in primary care in Zambia BMC Psychiatry 2011 11 75 10.1186/1471-244X-11-75 21542929 PMC3112078

[B33] Reid MC Fiellin DA O’Connor PG Harzardous and harmful alcohol consumption in primary care Arch Intern Med 1999 159 15 1681 10.1001/archinte.159.15.1681 10448769

[B34] Sunderland M Slade T Stewart G Andrews G Estimating the prevalence of DSM-IV mental illness in the Australian general population using the Kessler Psychological Distress Scale Aust N Z J Psychiatry 2011 45 10 880 889 10.3109/00048674.2011.606785 21875309

[B35] Patel V Kleinman A Poverty and common mental disorders in developing countries Bull World Health Organ 2003 81 8 609 615 14576893 PMC2572527

[B36] Lund C Breen A Flisher AJ Kakuma R Corrigall J Joska JA Swartz L Patel V Poverty and common mental disorders in low and middle income countries: A systematic review Soc Sci Med 2010 71 3 517 528 10.1016/j.socscimed.2010.04.027 20621748 PMC4991761

[B37] Araya R Rojas G Fritsch R Acuna J Lewis G Common mental disorders in Santiago, Chile: prevalence and socio-demographic correlates Br J Psychiatry 2001 178 228 233 10.1192/bjp.178.3.228 11230033

[B38] Williams DR Herman A Stein DJ Heeringa SG Jackson PB Moomal H Kessler RC Twelve-month mental disorders in South Africa: prevalence, service use and demographic correlates in the population-based South African Stress and Health Study Psychol Med 2008 38 2 211 220 17903333 10.1017/S0033291707001420 PMC2718686

[B39] Regier DA Farmer ME Rae DS Myers JK Kramer M Robins LN George LK Karno M Locke BZ One-month prevalence of mental disorders in the United States and sociodemographic characteristics: the Epidemiologic Catchment Area study Acta Psychiatr Scand 1993 88 1 35 47 10.1111/j.1600-0447.1993.tb03411.x 8372694

[B40] Carter GC Cantrell RA Victoria Zarotsky Haynes VS Phillips G Alatorre CI Goetz I Paczkowski R Marangell LB Comprehensive review of factors implicated in the heterogeneity of response in depression Depress Anxiety 2012 29 4 340 354 10.1002/da.21918 22511365

[B41] Seedat S Scott KM Angermeyer MC Berglund P Bromet EJ Brugha TS Demyttenaere K de Girolamo G Haro JM Jin R Karam EG Kovess-Masfety V Levinson D Medina Mora ME Ono Y Ormel J Pennell BE Posada-Villa J Sampson NA Williams D Kessler RC Cross-national associations between gender and mental disorders in the World Health Organization World Mental Health Surveys Arch Gen Psychiatry 2009 66 7 785 795 10.1001/archgenpsychiatry.2009.36 19581570 PMC2810067

[B42] Freeman M Nkomo N Kafaar Z Kelly K Factors associated with prevalence of mental disorder in people living with HIV/AIDS in South Africa AIDS Care 2007 19 10 1201 1209 10.1080/09540120701426482 18071963

[B43] Sullivan LE Fiellin DA O'Connor PG The prevalence and impact of alcohol problems in major depression: a systematic review Am J Med 2005 118 4 330 341 10.1016/j.amjmed.2005.01.007 15808128

[B44] Massak A Graham K Is the smoking-depression relationship confounded by alcohol consumption? An analysis by gender Nicotine Tob Res 2008 10 7 1231 1243 10.1080/14622200802163449 18629734

[B45] Lawrence D Mitrou F Zubrick SR Non-specific psychological distress, smoking status and smoking cessation: United States National Health Interview Survey 2005 BMC Public Health 2011 11 1 256 10.1186/1471-2458-11-256 21513510 PMC3107796

[B46] Ismail K Sloggett A De Stavola B Do common mental disorders increase cigarette smoking? Results from five waves of a population-based panel cohort study Am J Epidemiol 2000 152 7 651 657 10.1093/aje/152.7.651 11032160

[B47] Nel A Kagee A Common mental health problems and antiretroviral therapy adherence AIDS Care 2011 23 11 1360 1365 10.1080/09540121.2011.565025 22022846

